# Fat storage-inducing transmembrane proteins: beyond mediating lipid droplet formation

**DOI:** 10.1186/s11658-022-00391-z

**Published:** 2022-11-08

**Authors:** Gaiping Wang, Anqi Chen, Yu Wu, Danlin Wang, Cuifang Chang, Guoying Yu

**Affiliations:** State Key Laboratory Cell Differentiation and Regulation, Henan International Joint Laboratory of Pulmonary Fibrosis, Henan Center for Outstanding Overseas Scientists of Pulmonary Fibrosis, College of Life Science, Institute of Biomedical Science, Henan Normal University, Xinxiang, 453007 Henan China

**Keywords:** Fat storage-inducing transmembrane proteins (FITMs), Lipid droplets (LDs), Lipid metabolism, Endoplasmic reticulum stress, Metabolic disease

## Abstract

Fat storage-inducing transmembrane proteins (FITMs) were initially identified in 2007 as members of a conserved endoplasmic reticulum (ER) resident transmembrane protein gene family, and were found to be involved in lipid droplet (LD) formation. Recently, several studies have further demonstrated that the ability of FITMs to directly bind to triglyceride and diacylglycerol, and the diphosphatase activity of hydrolyzing fatty acyl-CoA, might enable FITMs to maintain the formation of lipid droplets, engage in lipid metabolism, and protect against cellular stress. Based on the distribution of FITMs in tissues and their important roles in lipid droplet biology and lipid metabolism, it was discovered that FITMs were closely related to muscle development, adipocyte differentiation, and energy metabolism. Accordingly, the abnormal expression of FITMs was not only associated with type 2 diabetes and lipodystrophy, but also with cardiac disease and several types of cancer. This study reviews the structure, distribution, expression regulation, and functionality of FITMs and their potential relationships with various metabolic diseases, hoping to provide inspiration for fruitful research directions and applications of FITM proteins. Moreover, this review will provide an important theoretical basis for the application of FITMs in the diagnosis and treatment of related diseases.

Fat storage-inducing transmembrane proteins (FITM1/2, FITMs) [also known as fat-inducing transcripts (FIT1/2)], were first identified by Kadereit and colleagues in late 2007, and are considered as two key genes involved in lipid droplet (LD) formation [1]. FITMs belong to a conserved gene family encoding endoplasmic reticulum (ER) resident transmembrane proteins, which are extensively found in various eukaryotes. When transiently expressed in the leaves of *Nicotiana benthamiana* or suspension cells of *Nicotiana tabacum*, Cai et al. found that FITM2 was specifically localized in the ER and was often concentrated at ER-LD junction sites [2], and that the ER-LD junction sites were proposed to function as a “vent” for the release of stored lipids from within the ER bilayer where nascent LDs were formed [3]. FITM1 and FITM2 are highly expressed in oxidative tissues including heart and skeletal muscle, and recent studies have demonstrated that the FITM family not only mediates lipid droplet formation and budding, but also participates in lipid metabolism and protects against cellular stress [4, 5]. Concerning the vital role of FITMs in lipid droplet biology and lipid metabolism, dysregulation of FITMs expression might result in lipid homeostasis-associated disorders or diseases. Accordingly, FITMs have been discovered to be closely related to the occurrence and development of type 2 diabetes, lipodystrophy, cardiac disease, and hepatocellular carcinoma, etc., and several researchers have even claimed that FITM proteins may serve as potential therapeutic targets for nonalcoholic fatty liver diseases (NAFLD) [6]. This review focuses on our current knowledge concerning the structure, distribution, expression regulation, and functions of FITMs, as well as their probable correlations with various metabolic diseases. These elucidations may facilitate future research directions and applications for FITM proteins, while providing an important theoretical basis for their use in the diagnosis and treatment of related diseases.

## Structure and distribution of FITMs

**Fig. 1:**
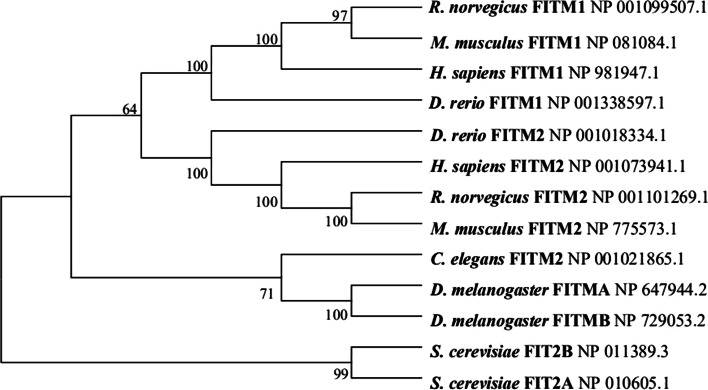
Sequence alignments of FITM orthologs in multiple model species. Cladogram generated with ClustalW showing the amino acid sequence homologies between FITM proteins. Accession numbers for each FITM protein are indicated next to the cladogram

In 2007, Kadereit et al. discovered two genes, and designated them as FIT1 and FIT2 (fat-inducing transcripts), which are also known as FITM1 and FITM2 (fat storage-inducing transmembrane proteins), confirming that they can induce the accumulation of lipid droplets [1]. FITM2 is an ancient member of the FITM gene family, and one of its homologs (SCS3) was first found in *Saccharomyces cerevisiae* [7], while the FITM1 homolog has long been found in zebrafish [1]. To date, hundreds of FITM orthologs have been identified from various species in the GenBank database. When comparing FITM family orthologs from different model species, FITM1 and FITM2 orthologous sequences have been found in mammals and several other organisms including yeast, drosophila, and zebrafish, whereas only a single FITM2 gene could be identified in *Caenorhabditis elegans*. The sequences of FITM family orthologs in non-vertebrates exhibited a lower homology than those of vertebrates (Fig. 1).

Both FITM1 and FITM2 genes encode proteins of over 200 amino acids and have 50% similarity to each other in terms of amino acid sequences. FITM1 and FITM2 do not have homology to known proteins or protein domains found in any species; thus, FITM genes comprise a unique gene family [1]. The human *FITM1* gene is located at 14q12, while the mouse *Fitm1* gene is located at 14C3. Meanwhile, they have similar gene structures, consisting of two exons and one intron, and encoding proteins of 292 amino acids. The human *FITM2* gene is located at 20q13.12, while the mouse *Fitm2* gene is located at 2H3. Both encode proteins of 262 amino acids. However, their genetic structures differ significantly as the human *FITM2* gene comprises two exons and one intron, whereas the mouse *Fitm2* gene consists of four exons, with the smallest fourth exon being only 7 bp in length. Concerning the spatial structures of these proteins, Gross et al. found that FITM proteins had six transmembrane domains, with both N- and C-termini localized to the cytosol. This is based on the predicted topological models for the FITM family using N-glycosylation site mapping and indirect immunofluorescence techniques [8]. Further protein alignments revealed that the FITM sequences contained the active site of lipid phosphatase/phosphotransferase (LPT) enzymes [9].

The distribution of FITM1 and FITM2 is significantly different in a variety of tissues. Mouse FITM1 mRNA was highly expressed in heart and skeletal muscle, but at lower levels in the liver, kidney, and testes, while FITM1 proteins were detected primarily in skeletal muscle, with lower levels in the heart [1]. Mouse FITM2 mRNA and proteins were ubiquitously detected at the highest levels in both white and brown adipose tissues. Furthermore, mouse FITM2 mRNA exhibited strong expression in the heart and skeletal muscle [1]. An examination of human tissues showed that FITM1 was primarily expressed in heart and skeletal muscle, whereas FITM2 was ubiquitously expressed in all detected tissues, with the highest levels in heart and skeletal muscle, kidney, pancreas, and lung [1]. Generally, FITM1 is primarily expressed in heart and skeletal muscle, while FITM2 is highly expressed in white and brown adipose tissues.

## Regulation of FITMs expression

**Fig. 2:**
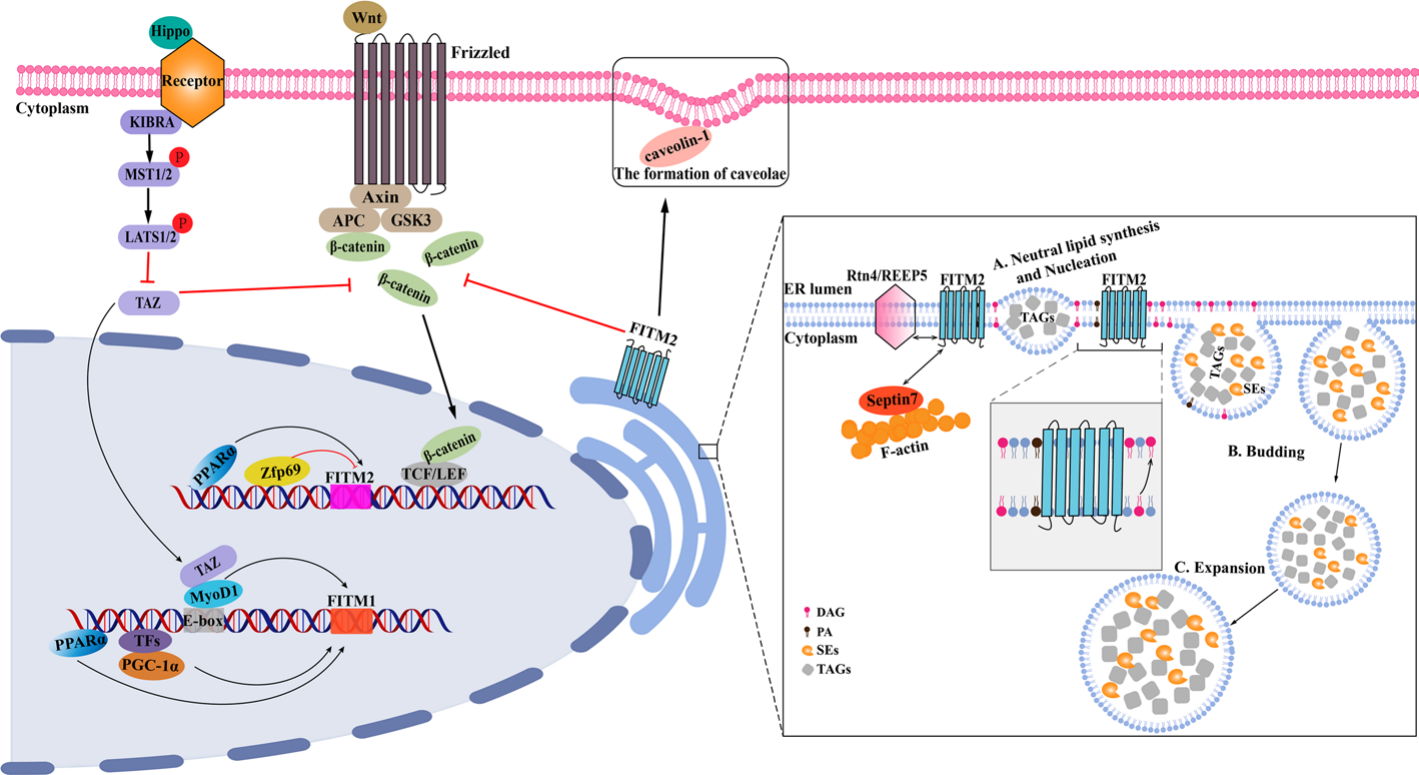
Schematic model of FITM expression regulation and their functions primarily in the formation and budding of LDs (modified mainly according to references [6, 20, 30, 47]). The expression of FITM1 can be upregulated by MyoD1, PPARα, and PGC-1α, while MyoD1 is activated by the Hippo signaling pathway. The expression of FITM2 is increased by PPARα, and decreased by Zfp69. The overexpression of FITM2 leads to the inhibition of the Wnt/β-catenin pathway and stimulates caveolae formation by regulating the expression of caveolin-1. The process of de novo LD biosynthesis is divided into three major steps: **A** Neutral lipid synthesis and nucleation; **B** Budding; **C** Expansion. During this process, FITM2 might help to concentrate TAG between the leaflets of the endoplasmic reticulum, and then promote the formation of an oil lens structure, which is the characteristic of the nucleation step. In addition, FITM2 maybe participate in reducing the DAG level in the cytoplasmic leaflets, and then facilitate the budding of lipid droplets. Furthermore, FITM2 could bind to cytoskeletal protein Septin7 and interact with ER tubule-forming proteins Rtn4 and REEP5 during nascent LD formation. *PGC-1α* peroxisome proliferator-activated receptor-gamma coactivator 1 alpha, *PPARα* peroxisome proliferator activated receptor alpha, *MyoD1* myogenic differentiation 1, *TAGs* triacylglycerols, *DAGs* diacylglycerols, *SEs* sterol esters, *PA* phosphatidic acid

In primary human skeletal muscle cells, the transcriptional coactivator peroxisome proliferator-activated receptor-gamma coactivator 1 alpha (PGC-1α) was able to stimulate the expression of the mRNA and protein levels of FITM1, and enhanced the formation of smaller lipid droplets, with only a modest increase in the triacylglycerol (TAG) content in oleate-incubated skeletal muscle cells [10]. Further analysis revealed that the MyoD1 transcription factor promoted FITM1 transcription by binding to the E-box element in the core promoter region of FITM1 during C2C12 differentiation, suggesting that FITM1 was a novel target for MyoD1 during muscle development [11]. Compared with FITM1, FITM2 has received more attention over the last 20 years. Several researchers have found that the mRNA expression of FITM2 was significantly reduced in the liver when an inhibitory regulator Zfp69 was overexpressed in lean mice, indicating that Zfp69 may regulate the transcription of FITM2 in diabetes and lipid metabolism [12]. A transcriptomic analysis conducted by Gupta et al. identified FITM2 as a novel target and downstream effector for miR-212/132 anticardiotoxicity [13]. Moreover, PPARα is a lipid-activated transcription factor that plays a vital role in regulating lipid metabolism, and data from hepatocyte-specific PPARα^−/−^ mice further supported that the FITM family might represent the direct target genes of PPARα [14]. The above series of studies illustrate that the expression of FITM family genes can be regulated by multiple upstream transcription factors including PGC-1α, MyoD1, and PPARα, where FITM1 is always regulated by transcription factors involved in muscle development and energy metabolism, while FITM2 is regulated by those associated with lipid metabolism (Fig. 2, left).

In addition, Fornes et al. found that mice injected with dihydrotestosterone, following 4–10 weeks of a high-glycemic diet, gained more liver weight on GD18.5 and had higher FITM1 mRNA expression compared with normal female mice [15], providing evidence for the relationship between the expression of FITM1 and diet. Recently, Zhang revealed that the vitrification temperature and cryoprotectant concentration may influence FITM2 gene expression in immature bovine oocytes after vitrification [16]. Moreover, it was shown that the FITM2 expression level was significantly overexpressed in 24 h dark-exposed zebrafish larvae [17]. All these studies suggest that the expression of FITM genes is influenced by both environmental stimuli and diet. Nevertheless, the signaling pathway or molecular mechanisms of FITM expression affected by environmental stimuli and diet remain unclear, requiring further investigation.

## Functions of FITM proteins

### FITMs maintain the formation of lipid droplets

Several studies have shown that FITMs are a class of endoplasmic reticulum proteins that participate in the formation and assembly of lipid droplets. When Kadereit et al. first identified the functions of the FITM family, they applied radiolabeled glycerol as a precursor for triglyceride (TG) to label newly synthesized TG, finding that human HEK293 cells expressing FITM1 or FITM2 contained similar amounts of labeled synthesized TG at all indicated time points (before 6 h) in comparison with mock-transfected cells. Subsequently, HEK293 cells were transfected with FITM1 or FITM2 and treated for 3 h with radiolabeled glycerol, after which lipid droplets were isolated and radioactively TG-quantified. The results showed that the quantity of labeled, newly synthesized TG in lipid droplets was four- to sixfold higher than that in the control cells, suggesting that FITM1 and FITM2 did not affect TGs biosynthesis, but rather partitioned TGs into lipid droplets [1]. Similarly, as two homologs of the FITM gene family in yeast cells, the transient expressions of SCS3 or YFT2 induced the appearance of LDs in HEK293 cells, which is similar to the findings on the overexpression of human and mouse FITM genes [4]. Additionally, a study employing primary adipocyte precursors of mouse white and brown adipose tissues also revealed that FITM2 could regulate the number and size of lipid droplets without impacting the total cellular TGs or TGs biosynthesis [18].

Lipid droplets (LDs) are organelles that store lipids consisting of a neutral lipid core (triacylglycerols and sterol esters) [19], where the process of de novo LD biosynthesis is typically divided into three major steps: A: Neutral lipid synthesis and nucleation; B: Budding; C: Expansion [20]. Regarding the specific roles of FITM proteins in LD formation, Gross et al. found that FITMs could bind directly to TGs and DAGs in vitro, where the binding capacity was essential for the FITM-mediated formation and assembly of LDs. This was because FITM gain-of-function mutants of HEK293 cells were capable of binding more TGs and forming larger LDs in contrast to normal cells, whereas these mutants with FITM1 or FITM2 partial loss-of-function had a significantly reduced ability to bind TGs; thus, they produced smaller LDs [21]. Additionally, the ability of FITM1 to bind TGs and DAGs was weaker than FITM2, resulting in smaller LDs [21]. At the onset of LD biosynthesis, TGs/SEs are synthesized and dispersed between the two leaflets of the ER membrane [22]. With the increase in their concentration, TGs/SEs eventually coalesce and form an oil lens, which is the characteristic of the nucleation step of de novo LD biogenesis [23]. During this process, Goh et al. predicted one possible model of FITM function was that FITM proteins play a role in the initial local accumulation of TGs within the leaflets of the ER owing to the TG-binding capacity of FITM proteins [6]. However, inconsistent with the hypothesis proposed by Goh et al., Zoni et al. found that Seipin was able to cluster TG molecules, and then generated ER sites with a specific lipid composition that in turn recruits FITMs and perilipins; thus, the formation of an oil lens structure could be facilitated [24]. Nevertheless, both of these studies indicate that FITM proteins are vital during the nucleation step of LD biogenesis. However, no study has identified the direct relationship between the TG-binding capacity of FITMs and the local accumulation of TGs within the ER leaflets. That is to say, it remains elusive whether and how the TG-binding capacity of FITMs function in the accumulation of TGs within the ER leaflets, which may be the aim of future studies. In addition, it was also observed that FITM2 could bind to the cytoskeletal protein Septin7, and interacted with ER tubule-forming proteins Rtn4 and REEP5, since the depletion of ER tubule-forming proteins or Septins delayed nascent LD formation [25]. As a result, cytoskeleton interactions also play an essential role in this process (Fig. 2, right).

With further understanding of the mechanisms behind lipid droplet budding from the ER, FITM proteins are revealed to be essential for stimulating the budding of nascent LDs from the ER. The elimination or reduction of SCS3 and YFT2 negated the capacity of LDs to bud from the ER; thus, LDs remained within the ER membrane [26, 27]. A recent study employing an in vitro model system suggested that the LD budding direction was determined by the tension asymmetry between the monolayers that enclose the droplet, therefore, budding typically occurred toward the side of lower monolayer tension [28]. Similarly, the surface tension facing the ER lumen is always higher than that facing cytoplasmic membrane, inducing nascent lipid droplets to bud into the cytoplasm [29]. Additionally, the many DAGs contained within the cytoplasmic leaflets of pre-LDs increase the monolayer tension on the cytoplasmic side, favoring the embedded LDs and preventing their budding into the cytosol [20]. Nevertheless, Choudhary et al. found that DAG levels were significantly higher in purified ER membranes from cells lacking FITM2 proteins than those from wild-type cells, and FITM2 was always enriched at LD generation sites when LD biogenesis was induced, concluding that FITM2 proteins may promote the emergence of LDs from the ER by reducing DAG levels at LD generation sites [30]. Subsequently, another study demonstrated that FITM2 was a fatty acyl-coenzyme A (CoA) diphosphatase, considered FITM2 to be the only acyl-CoA hydrolase in the ER lumen until now, and then proposed an attractive hypothesis that the cleavage of the acyl-CoA phosphoanhydride bond might be coupled to conformational changes inducing the active pumping of neutral lipids across the membrane; such activities could be important toward reducing DAG levels in the vicinity of LD budding at the cytosolic leaflet to facilitate budding [31]. Nevertheless, the mechanistic link between enzymatic activity of FITM2 and the reduction of DAG levels in the vicinity of LD budding requires further investigation.

Briefly, FITMs might maintain normal LD formation primarily through two major steps of de novo LD biosynthesis including the nucleation step and the budding step (Fig. 2). Clearly, FITMs can bind TGs and DAGs, and have hydrolysis enzymatic activity. However, there are some hypotheses and speculations about their specific roles during LD biosynthesis, which need to be further investigated.

### FITMs are involved in lipid metabolism

Since FITMs are intimately associated with the formation of LDs, which always play central roles in lipid and energy metabolism, FITMs may be involved in lipid metabolism. Therefore, numerous studies have also examined whether FITMs could be involved in the biosynthesis of TGs. For example, Kadereit et al. initially discovered that the knockdown of FITM2 expression in 3T3-L1 adipocytes generated a dramatic reduction in lipid droplets and a significant decrease in the total cellular TGs. Thus, they proposed that an inhibition in the ability to generate lipid droplets caused a partial feedback inhibition in TGs biosynthesis [1]. Furthermore, Becuwe et al. found that the mRNA expression of genes involved in TGs synthesis (e.g., LIPIN1, DGAT1, and DGAT2) were significantly reduced in human FITM2 knockout SUM159 cells, and predicted that this might be the result of an adaptation to long-term FITM2 deletion [31]. In contrast, HEK293 cells expressing an enhanced FITM2 variant exhibited a fivefold increase in both LD number and size, but only a 1.8-fold increase in cellular triglycerides compared with wild-type FITM2 [8]. Subsequently, Cai et al. found that the ectopic overexpression of mouse FITM2 greatly increased the number and size of lipid droplets in both leaf and seed tissues of *Arabidopsis thaliana*, whereas enhanced the oil content by 13% in some FITM2-expressing *Arabidopsis thaliana* seeds [2]. The above-mentioned two studies can be explained by the observation that FITM2 does not mediate triglyceride biosynthesis, but rather the redistribution or partitioning of cellular triglycerides into lipid droplets. The half-lives of triglycerides within lipid droplets may be longer than in cellular membranes, contributing to modest increases in cellular triglycerides in response to FITM overexpression [8]. Moreover, mice with skeletal muscle-specific overexpression of FITM2 exhibited a sevenfold increase in intramyocellular total triglycerides, whereas the activity of diacylglycerol acyltransferase, as a rate-limiting enzyme in the final step of TGs synthesis, was unchanged in muscles relative to WT muscle. This can further support the previous finding that FITM2 does not mediate triacylglycerol biosynthesis [32]. Thus, it is quite evident that FITMs are involved in lipid formation and storage, whereas the increase in TG is more akin to a downstream effect of additional LD budding from the ER in response to FITM overexpression. Therefore, until now, the prevailing view is still that FITMs are not involved in TG synthesis but generate an important role in LD formation. Nevertheless, several researchers also acknowledged that FITM2 could associate with domains involved in both TAG synthesis and nascent LD biogenesis, and potentially enhance a general coordination of neutral lipid synthesis and LD formation [2]. Nevertheless, the coordinating role of the FITM family in neutral lipid synthesis and LD formation requires further investigation.

With regard to the correlation between FITMs and phospholipids metabolism, Hosaka et al. first speculated that SCS3 was involved in regulating inositol synthesis, but they did not quantify levels of inositol phospholipids in SCS3 mutants or provide direct evidence that SCS3 regulated this pathway [7]. However, Gaspard et al. later demonstrated that SCS3 could promote the expression of INO1 (the rate-limiting enzyme in the synthesis of phosphatidylinositol) in yeasts [33]. Consistently, another study also discovered that SCS3 could properly regulate the transcription of INO1 and other genes involved in lipid metabolism [4]. Except for potentially facilitating LD budding as a fatty acyl-CoA diphosphatase, the catalytic residues in FITM2 were also demonstrated to be crucial for inositol prototrophy in yeasts in vivo [31], further confirming the role of FITM2 in inositol synthesis. More importantly, the substrate for FITM2, acyl-CoA, is located in the ER lumen, and the acyl-CoA hydrolysis activity of FITM2 appears to be localized to the ER lumen [31], indicating that FITM2 could be easier to act on its substrate. Thereafter, the study proposed one possibility that FITM2-mediated hydrolysis of acyl-CoAs helps prevent the build-up of fatty acyl-CoAs on the luminal leaflet of the ER, and then contributes to maintaining normal phospholipid balance between the two ER leaflets [31]. Therefore, it is clear that the FITM family may be involved in phospholipid metabolism. However, more direct or stronger evidence needs to be provided in the future.

The distinct distribution of FITMs within tissues and their capacity to bind TGs raised the notion that FITM1 is vital for the generation of small LDs involved in the rapid mobilization of TAGs during increased ATP demand [32]. By contrast, FITM2 was primarily involved in the generation of large LDs for the long-term storage of TAGs [32]. Mice with skeletal muscle-specific overexpression of FITM2 were reported to exhibit decreased fatty acid oxidation, increased utilization of branched-chain amino acids, and enhanced glucose uptake, as well as decreased cellular ATP levels in skeletal muscle [32], which could preliminarily imply a correlation between FITMs and energy metabolism. Additionally, the reaction products of the FITM2 enzyme (acyl 4′-phosphopantetheine and adenosine-3′,5′-bisphosphate) were linked to important metabolic processes including CoA and energy metabolism [34]. Therefore, with the rapid growth of interest in FITM family, FITMs have been gradually related to the metabolism of several substances associated with energy supply and storage.

### FITMs protect against cellular stress

Proper ER functionality is essential for cells; however, various conditions can interfere with its operation, leading to a build up of proteins, referred to as ER stress [35]. Unfolded protein response (UPR) is caused by the build up of misfolded proteins in the ER, which is the classical signaling pathway for ER stress [36, 37]. UPR might be activated by specific conditions including dithiothreitol (DTT), tunicamycin (Tm), and low concentrations of inositol [38]. In yeasts, Yap et al. demonstrated that SCS3 was vital for the activation of stress-induced UPR caused by Tm, and for the viability of yeast in the absence of UPR transducer inositol requiring enzyme 1 (Ire1) [27]. Furthermore, Moir et al. showed that SCS3 and YFT2 deletion strains were significantly compromised for UPR, concluding that they were required for the normal response to and recovery from ER stress under inositol depletion and DTT [4]. Thus, as one of the downstream UPR target genes, FITM2 may respond to ER stress primarily by enhancing its expression. In contrast, Becuwe et al. utilized mammalian cells, revealing that the knockout of FITM2 resulted in dramatically altered ER morphology and the elevated expression of ER stress markers (ATF3, BIP, and CHOP) compared with wild-type cells. However, the expression of wild-type FITM2 in the knockout of FITM2 cell lines completely reversed the aberrant ER morphology phenotype and the upregulated expression of ER stress markers [31]. Therefore, the role of FITM2 in ER stress still appears significantly divergent, which may be caused by the presence of the inverse feedback regulatory role of increased FITM2 during normal responses to ER stress.

Concerning the potential relationship between FITMs and ER morphology, Becuwe et al. revealed that the absence of FITM2-mediated hydrolysis of acyl-CoAs in FITM2-knockout cells led to excess luminal acyl-CoA, which could disrupt ER membranes, possibly by acting as detergents to destabilize membranes or by altering phospholipid balance, indicating that the enzymatic activity of FITM2 could indirectly influence ER morphology and stress [31]. Another possible reason is that the knockout of FITM2 results in a defect in the ability to package TGs in lipid droplets, and the inability to bud lipid droplets likely significantly disrupts the ER [23]. Nevertheless, ER stress may be an indirect result of FITM2 deletion, and the mechanistic linkages between FITM2 deficiencies and ER stress require further verification. In addition to involvement in ER stress response, Nguyen et al. found that FITM2 gene deletion enhanced the sensitivity to oxidative stress when utilizing FITM2-deficient Candida mutants to detect their susceptibility to H_2_O_2_, amphotericin B, and azole [39], which is most likely consistent with the finding in terms of FITM2 deletion in ER stress.

In summary, it is certain that FITM2 deletion results in abnormal lipid metabolism and equally leads to ER stress. Additionally, Han et al. found that ER stress induced the higher expression of lipid-related genes leading to abnormal lipid metabolism [40]. As a result, the cause–effect relationships among FITM2, abnormal lipid metabolism, and ER stress need to be sufficiently clarified, including whether the absence of FITMs causes ER stress, thereby generating abnormal lipid metabolism.

## FITMs are involved in various diseases

### FITMs and lipid metabolic dysfunction

The process of lipid droplet formation often exerts an important role in both cellular physiology and disease. Given that FITMs play a vital role in lipid droplet formation, as well as lipid metabolism, downregulated FITMs might generate lipid homeostasis-associated disorders or diseases. In *Caenorhabditis elegans*, FITM2 deficiency led to a significant reduction in intestinal lipid droplets and dramatic defects in muscle development, leading to the death of almost all homozygous FITM2 animals as larvae [41]. Similarly, tamoxifen-induced FITM2 deletion in the whole body of a mouse model was shown to cause lethal intestinal lesions and death within 2 weeks. This was owing to the postnatal knockdown of FITM2, inducing the absence of cytosolic LDs in the intestinal epithelial cells of mice. Thus, the intestinal bile acid transporters were transcriptionally dysregulated, after which the bile acids continued to accumulate within enterocytes [42]. Consequently, FITM2 deletion in the whole body typically led to animal death. However, Seco et al. found that fruit flies exhibited hearing impairment, locomotor defects, and abnormalities of the sensory system when employing RNA interference to knock down the single Drosophila FITM ortholog FITM2 [43]. Conversely, the tissue-specific knockout of FITMs may be not so severe, as some studies found that in response to it, the adipose tissue of mice exhibited a series of physiological and biochemical features (including lipodystrophy, insulin resistance, greater inflammation, and ER stress) [32, 44]. Rats with adipose tissue-specific knockout of FITM2 exhibited progressive adipose atrophy and metabolic dysfunction in white adipose tissue [18]. The overexpression of FITM2 in porcine intramuscular preadipocytes led to the upregulation of peroxisome proliferator-activated receptor γ and CCAAT enhancer binding protein-α proteins, and downregulation of the β-catenin protein, which can be partially neutralized by LiCl (an inhibitor specific for the Wnt/β-catenin pathway) [45]. The obtained results indicate that FITM2 promotes porcine intramuscular preadipocyte differentiation by inhibiting Wnt/β-catenin signaling.

After sequencing and exploring a consanguineous family from Pakistan, who were ascertained to have a novel deafness–dystonia syndrome with motor regression, ichthyosis-like features, and signs of sensory neuropathy, Seco et al. confirmed that the patient’s symptoms were caused by a mutation in the FITM2 gene. This provided the first independent evidence for the involvement of FITM2 in human disease [43]. Subsequently, Agrawal et al. discovered that the FITM2 protein was significantly decreased in subcutaneous and omental adipose tissue from diabetic patients in comparison with that of normal subjects. Further studies discovered that the partial loss of FITM2 proteins in primary human adipocytes attenuated their lipid storage capacities and induced insulin resistance. This demonstrated that FITM2 was correlated with human type 2 diabetes and essential for maintaining the functionality of healthy adipose tissues [44]. Consistent with these results, Cho et al. identified the presence of a susceptibility locus for type 2 diabetes on the FITM2 gene based on a meta-analysis of genome-wide association studies [46]. Nonalcoholic fatty liver diseases (NAFLD) are characterized by the accumulation of fat in the liver, which, if not effectively treated, can further deteriorate into cirrhosis, end-stage liver disease, or hepatocellular carcinoma. In view of the important roles of FITM proteins in lipid droplet biology, several researchers pointed out that FITM proteins might serve as potential therapeutic targets for NAFLD [6, 47]. Dewhurst et al. discovered a preliminary relationship between the expression of FITM1 and the occurrence of hepatic fibrosis using RNA-sequencing analysis [48]. However, no other findings can suggest the specific functions of FITMs in NAFLD, requiring further investigation. In summary, the absence of FITMs reduces cellular lipid storage capacities, while their overexpression often promotes adipose differentiation and the over-accumulation of lipid droplets. As a result, the abnormal expression of this protein is often implicated in a variety of lipid metabolic dysfunction-related diseases.

### FITMs and other diseases

FITMs are typically highly expressed in heart muscle; thus, it is not surprising that the dysfunction of FITMs can lead to the occurrence of cardiac diseases. It was clear that the lipid content of myocardiocytes was increased in failing hearts [49], and the deletion of FITM2 was found to generate protective effects against chronic heart failure due to volume and pressure overload [50]. From the perspective of energy metabolism, heart failure was accompanied by the reduced utilization of fatty acids and ATP levels [51, 52]. Nevertheless, whether the deletion of FITM2 affects energy metabolism in failing hearts remains a vital question to be addressed. Furthermore, the miR-212/132 family might prevent doxorubicin-induced cardiac atrophy and cardiotoxicity. The overexpression of FITM2 (a novel target of miR-212/132) enabled the partial reversal of the effects of miR-212/132, providing a new concept for limiting adverse doxorubicin-mediated cardiac effects [13]. Increasing studies on FITMs may provide new insights and approaches for the treatment of cardiac diseases in the future.

Currently, the roles of the FITM family in cancer pathogenesis have received increased attention. Interferon-γ (IFN-γ) resistance is usually considered to be an intrinsic conserved cancer mechanism of cytotoxic T lymphocytes (CTLs) immune evasion for cancer cell. Lawson et al. found that FITM2 was required for maintaining the fitness of several types of cancer cells following exposure to CTL-produced IFN-γ [53]. Additional research indicated that FITM2 promoted caveolae formation by regulating the expression of caveolin-1, thus stimulating hepatocellular carcinoma (HCC) migration. Furthermore, the high intratumoral expression of FITM2 was related to poor HCC prognosis, which might be applied in the development of new adjuvant therapies [54]. These two studies indicated that FITM2 facilitated the survival of some cancer cells or the development of HCC. In contrast, Yang et al. identified FITM2 as one of the key differentially expressed mRNAs in right-sided colon adenocarcinoma (RSCOAD) compared with left-sided colon adenocarcinoma (LSCOAD) by TCGA integration analysis, implying the complex regulatory role of FITM2 in different pathological types of colon cancer [55]. The research methods and cancer models used in the above studies varied dramatically, which might translate to the conflicts in the role of FITM2 in the development of cancer. Consequently, further research is required to confirm the essential functions of FITM2 in cancer pathogenesis. As for FITM1, Chen et al. found that FITM1 was significantly downregulated, which might trigger carcinogenesis and the progression of non-viral HCC, and also identified it as a potential tumor suppressor gene in non-viral HCC pathogenesis [56]. Another analysis employing 179 pancreatic cancer samples and 171 normal pancreatic tissue samples on the GEPIA platform revealed that FITM1 was deferentially downregulated in pancreatic cancer, and elevated FITM1 expression was associated with prolonged overall survival in pancreatic cancer patients [57]. Both the above-mentioned studies elucidate the inhibitory role of FITM1 in carcinogenesis. However, it remains uncertain whether FITM1 might promote some cancers as does FITM2. Moreover, abnormal energy metabolism is one of the hallmarks of cancer and is closely linked to therapy resistance, and it deserves further study whether the involvement of FITMs in cancers is owing to their roles in lipid or energy metabolism; as a result, further research may lead to new findings on the role of FITMs in metabolic process of cancer pathogenesis.

## Summary and prospects

Taken together, this study reviews the structure, distribution, expression regulation, and main functions of FITM proteins and their association with a variety of metabolic dysregulations or diseases. FITMs are initially observed to be involved in lipid droplet formation. However, subsequently, their functions have been found to go far beyond the partitioning of TG into LDs. Current studies have demonstrated that FITMs not only affect lipid metabolism homeostasis, but also protect against cellular stress, even though the cause–effect relationships between FITM2, abnormal lipid metabolism, and ER stress remain uncertain. Accordingly, FITMs proteins are found to be involved in various metabolic diseases, including type 2 diabetes, lipodystrophy, cardiac diseases, and several types of cancers. Nevertheless, the mechanisms underlying the effects of FITMs in various diseases are not well understood. More studies on FITMs may provide new concepts and strategies for treating these diseases in the future. The liver is an important organ involved in lipid metabolism. However, there are few studies on the relationships between the FITM family and liver diseases including NAFLD, nonalcoholic steatohepatitis (NASH), liver cirrhosis, and HCC. In the past, NAFLD research has limited its focus on TG accumulation in hepatocytes [58]. In fact, TGs are always accumulated and packaged within lipid droplets, which represents a defining feature of fatty liver disease, and an aspect of biology that is only now coming into full view. Therefore, an improved elucidation of the FITM family pertaining to the synthesis of LDs, will provide exciting new strategies for interventions to address the development of complications arising from liver steatosis, while contributing to developing FITM proteins as potential therapeutic targets for various liver diseases.

## Data Availability

Not applicable.

## Abbreviations

FITMs: Fat storage-inducing transmembrane proteins
ER: Endoplasmic reticulum
LDs: Lipid droplets
TAG: Triacylglycerol
LPT: Lipid phosphatase/phosphotransferase
TG: Triglyceride
CoA: Fatty acyl-coenzyme A
UPR: Unfolded protein response
DTT: Dithiothreitol
Tm: Tunicamycin
NAFLD: Nonalcoholic fatty liver diseases
NASH: Nonalcoholic steatohepatitis
HCC: Hepatocellular carcinoma
